# The safety and accuracy of radiation-free spinal navigation using a short, scoliosis-specific BoneMRI-protocol, compared to CT

**DOI:** 10.1007/s00586-025-09151-x

**Published:** 2025-07-21

**Authors:** Peter P.G. Lafranca, Yorck Rommelspacher, Sebastian G. Walter, Sander P.J. Muijs, Tijl A. van der Velden, Yulia M. Shcherbakova, Rene M. Castelein, Keita Ito, Peter R. Seevinck, Tom P.C. Schlösser

**Affiliations:** 1 Department of Orthopedic Surgery, University Medical Center Utrecht, Utrecht, The Netherlands; 2 Department of Spine Surgery, Krankenhaus der Augustinerinnen, Cologne, Germany; 3 Department of Orthopedic Surgery, University Hospital Cologne, Cologne, Germany; 4 Department of Radiology, Image Sciences Institute, University Medical Center Utrecht, Utrecht, The Netherlands; 5 MRIguidance B.V., Utrecht, The Netherlands; 6 Department of Biomedical Engineering, Eindhoven University of Technology, Eindhoven, The Netherlands

**Keywords:** Radiation-free, MRI, AI-generated, Synthetic CT, Navigation, Pedicle screws

## Abstract

**Purpose:**

Spinal navigation systems require pre- and/or intra-operative 3-D imaging, which expose young patients to harmful radiation. We assessed a scoliosis-specific MRI-protocol that provides T2-weighted MRI and AI-generated synthetic-CT (sCT) scans, through deep learning algorithms. This study aims to compare MRI-based synthetic-CT spinal navigation to CT for safety and accuracy of pedicle screw planning and placement at thoracic and lumbar levels.

**Methods:**

Spines of 5 cadavers were scanned with thin-slice CT and the scoliosis-specific MRI-protocol (to create sCT). Preoperatively, on both CT and sCT screw trajectories were planned. Subsequently, four spine surgeons performed surface-matched, navigated placement of 2.5 mm k-wires in all pedicles from T3 to L5. Randomization for CT/sCT, surgeon and side was performed (1:1 ratio). On postoperative CT-scans, virtual screws were simulated over k-wires. Maximum angulation, distance between planned and postoperative screw positions and medial breach rate (Gertzbein-Robbins classification) were assessed.

**Results:**

140 k-wires were inserted, 3 were excluded. There were no pedicle breaches > 2 mm. Of sCT-guided screws, 59 were grade A and 10 grade B. For the CT-guided screws, 47 were grade A and 21 grade B (*p* = 0.022). Average distance (± SD) between intraoperative and postoperative screw positions was 2.3 ± 1.5 mm in sCT-guided screws, and 2.4 ± 1.8 mm for CT (*p* = 0.78), average maximum angulation (± SD) was 3.8 ± 2.5° for sCT and 3.9 ± 2.9° for CT (*p* = 0.75).

**Conclusion:**

MRI-based, AI-generated synthetic-CT spinal navigation allows for safe and accurate planning and placement of thoracic and lumbar pedicle screws in a cadaveric model, without significant differences in distance and angulation between planned and postoperative screw positions compared to CT.

## Introduction

The use of navigation has proven to improve accuracy of screw placement and reduce complications [1]. Especially in pediatric patients with spinal deformities, the three-dimensional (3-D) orientation of the pedicles may be severely altered. Pedicle screw malposition can lead to major complications like spinal cord or nerve root injuries or perforations of surrounding structures. These complications are rare, but can be devastating. Using the freehand technique, screw misplacements rates range from 1.5% to as high as 29% [2–4]. Image-guided navigation shows significantly lower pedicle breach rates and returns to the operating room compared to freehand pedicle screw instrumentation [1, 5, 6].

All currently available spinal navigation techniques require reference input from pre- and/or intra-operative 3-D imaging. Most often this includes preoperative computed tomography (CT) scans and intraoperative 2-D or 3-D fluoroscopy. This exposes patients and OR staff to harmful ionizing radiation. Treatment of pediatric patients with spinal deformities is already radiation-heavy, especially for surgically treated patients [7]. This is caused for a large part by peri-operative imaging. Cumulative radiation exposure in adolescent idiopathic scoliosis (AIS) patients has been shown to result in a 5-times increased cancer incidence compared to the general population [8]. 1 in every 1000 patients treated with intraoperative navigation develops an iatrogenic malignancy [9]. Based on estimates by Cool et al. (2023) in patients treated with low-dose planning CT and 3-D printed guides, the CT delivered a median effective dose of 0.942mSV, 51% of the total 1.841mSV [10]. Therefore, there is great need to reduce radiation exposure to as low as reasonably possible, while keeping advantages of image-guided spinal navigation surgery.

Recently, we developed a short, 14-minute (11-minute without cervical spine imaging), scoliosis-specific and radiation-free MRI protocol which provides both MRI and 42 centimeter (cranial– caudal) MRI-based AI-generated synthetic CT (sCT) images in one examination, using commercially available software (BoneMRI V1.7, MRIguidance BV, Utrecht, NL). This MRI protocol has high precision, reliability and agreement for scoliosis-specific measurements and covers the entire thoracic and lumbar (scoliotic) spine [11]. Earlier case series on sCT showed it can be used for morphological assessments in a similar manner as regular CT [12–17].

In this investigator-initiated cadaveric study, we aim to test the feasibility and safety of a workflow that combines existing spinal navigation systems with preoperative MRI-based sCT compared to regular CT. The purpose of this cadaveric study is to determine whether synthetic CT-guided spinal navigation is feasible and shows significant differences compared to CT-guided navigation in terms of pedicle breach rate and screw position accuracy. We hypothesize that sCT images can be integrated within existing navigation software to perform navigated spinal surgeries without radiation exposure and that it is safe in terms of pedicle breaches and screw accuracy in this cadaveric experiment.

## Materials and methods

*Experimental setup and cadaveric imaging*

Five fresh-frozen cadavers with intact spines were obtained from the university anatomy department. For the body donation, consent was obtained. The specimens were aged 74–93 years old (mean 83.6), 1 female and 4 males. None showed signs of previous spinal surgery or anatomical anomalies. There were signs of normal aging. All cadavers were defrosted to 21 degrees Celsius and scanned with both high-resolution thin-slice CT and the in-house developed scoliosis-specific MRI scanning protocol of the thoracic and lumbar spine. CT data were acquired at a Philips IQon CT, with a resolution of 0.84 × 0.84 × 0.7 mm and tube voltage of 120KvP. MRI data were acquired at a Philips 1.5T Achieve dStream, with field of view (FOV) of 420 × 220 × 100 mm (FH/AP/LR) at a resolution of 0.625 × 0.625 × 1.0 mm in the sagittal orientation, using a 3-D Dual Echo Spoiled Gradient echo sequence (Flip angle: 10 degrees, TR: 7 ms, TEs: 2.1/4.2ms). The maximum FOV (cranial-caudal) of 42 cm was positioned such that the caudal end included the S1 endplate. For 3 cadavers vertebral levels T3 - L5 were visualized, for 1 cadaver T5 - L5 and 1 cadaver T6 - L5; a total of 140 pedicles. The total scanning time per cadaver to obtain 3-D sagittal T2-weighted MRI and BoneMRI was 11 min and 2 s. From the MRI, axial multi-planar reconstructions were made and manual correction of the imaging modality tags from MRI to CT was done to match in the input requirements of the surgical navigation systems. MRI scans were converted to synthetic CTs using commercially available software (BoneMRI V1.7, MRIguidance BV, Utrecht, NL).

*Preoperative screw planning*

**Fig. 1:**
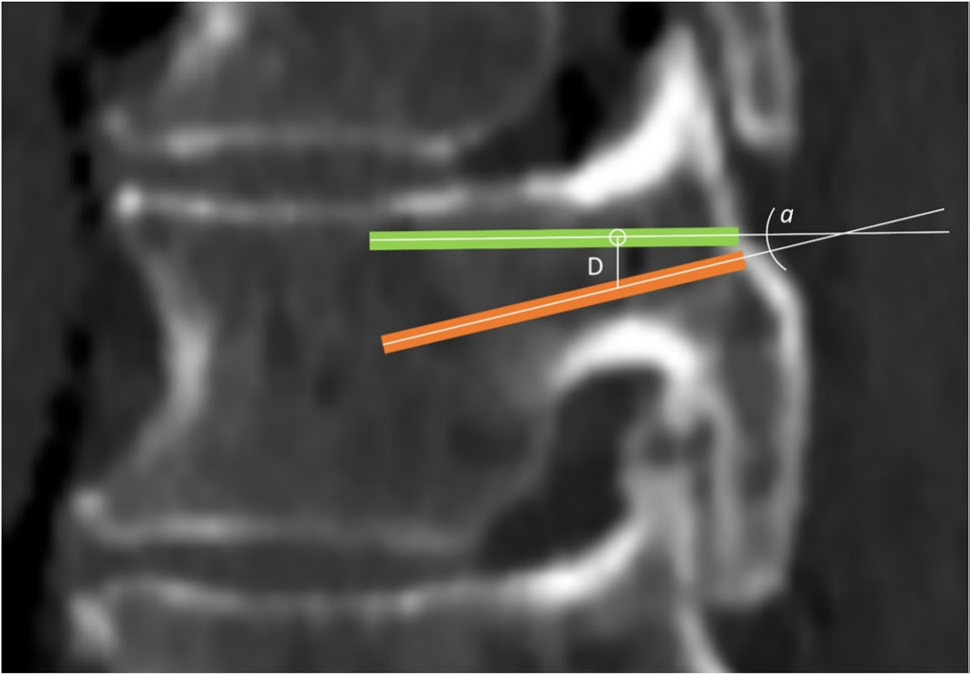
Representation of the maximum angle and distance. At one third of the planned screw (green trajectory), a virtual circle perpendicular to the screw was expanded till it touched the other screw axis (orange trajectory), and the distance (**D**) between these was measured. The maximum angle (**a**) between both screw trajectories was also calculated

Screws were planned on both CT and sCT at the same pedicle levels, and the coordinates of screw trajectories were saved. Each vertebra was registered in one fusion matrix, and maximum angulation and distance at the level of the pedicle (standardized at one third of total screw length) were compared between both modalities (Fig. 1).

*Surgical setup*

Cadavers were positioned prone on standard anatomy tables. After a midline posterior approach, the Kick^®^ navigation system (Brainlab, Munich, Germany) was used by four experienced spine surgeons for spinal navigation, using surface matching on each vertebral level by a 20-point cloud and the standard protocol for verification of the accuracy of registration. Randomization for CT/sCT, surgeon, and side was performed with a 1:1 ratio. Drilling was performed using the navigated drill guide in an optimal trajectory for a virtual screw. Screw thickness and length was standardized for each vertebral level: 4.5 × 35 mm at T3 – T4, 5.5 × 40 mm at T5 – T12 and 6.5 × 40 mm at L1 – L5. After drilling, 2.5 mm aluminum Kirschner wires (k-wires) were placed.

*Postoperative imaging and screw accuracy assessment*

A postoperative thin-slice CT scan was obtained, with similar properties as the preoperative CT-scan. A metal artifact filter was applied to the CT-scan (OMAR, Philips B.V., Eindhoven, The Netherlands). K-wire positions were visualized in 3-D. Over each k-wire, virtual screws with predefined sizes were placed by an independent, blinded researcher. Medial pedicle breaches of virtual screws were scored using the Gertzbein-Robbins classification (Table 1) [18]. After registration of each vertebra in one fusion matrix, the intraoperatively planned screw positions were compared with the real postoperative position by maximum 3-D angulation and distance at the level of the pedicle. The distance was defined as the length of a line perpendicular to the planned screw, intersecting with the postoperative screw (Fig. 1). Outliers with ≥ 10 mm distance between intraoperative and postoperative screw positions were evaluated by manual comparison of the images, to retrieve postoperative registration or labelling errors and not true navigation inaccuracy.

### Statistical analysis

Calculations were performed using Microsoft Excel 365 (Microsoft, Redmond, Washington, USA) and SPSS 29.0.1 for Windows (IBM, Armonk, NY, USA). The difference in pedicle breach rate was assessed with a chi-square test of independence. Both the angle and distance between planned and postoperative screws, as well as between the modalities, were compared using an independent samples t-test, with *p* < 0.05 considered as a significant difference.

**Table 1:** Gertzbein-Robbins classification [18]

Grade	Definition
Grade A	Screw is completely within the pedicle
Grade B	Pedicle cortex breach < 2 mm
Grade C	Pedicle cortex breach > 2–4 mm
Grade D	Pedicle cortex breach > 4–6 mm
Grade E	Pedicle cortex breach > 6 mm

## Results

*Feasibility workflow*

**Fig. 2:**
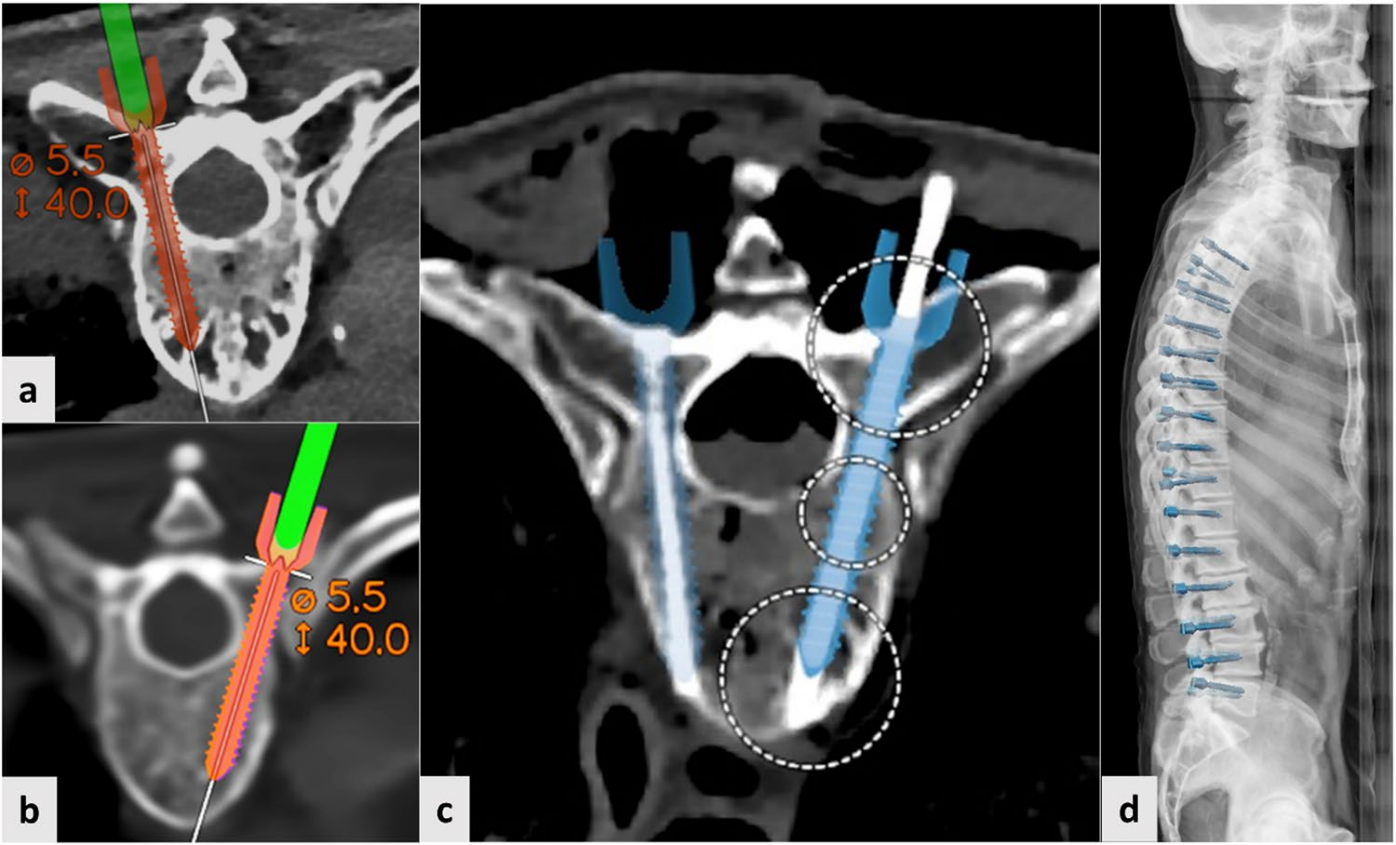
(**a**) Example of planned intraoperative screw position in thoracic vertebra on a CT-scan; (**b**) example of planned intraoperative screw position in thoracic vertebra on a sCT scan; (**c**) axial postoperative CT-scan with a virtual screw positioned over the k-wire. The dotted circles could be used to adapt the screw position to exactly overlay the k-wire (**d**) sagittal reconstruction of all postoperative screw positions in the navigation software

The integration of both CT and sCT scans in the navigation software was successful and surface matching was possible on all levels for both modalities. All surgeons reported that surface matching worked easily and accurately, without noticing clear differences between modalities. Average time for surface matching and subsequent pedicle screw placement was 4 min. Figure 2 shows examples of planned and postoperative screw trajectories.

*Preoperative planning accuracy*

It was possible to perform planning of screws on the sCT images in the navigation software. Surgeons reported no issues regarding planning. The distance and maximum angulation (± SD) between the 70 pedicle screws that were planned on sCT as well as CT, was 1.9 mm ± 1.5 mm and 6.9° ± 3.9°, respectively.

*Surgical screw placement accuracy*

137 screws were included for the safety and accuracy comparison between CT and sCT, three outliers (2 CT-guided, 1 sCT-guided) with > 10 mm difference were excluded. These outliers were caused by errors in the pre- to postoperative semi-automatic registration during the image processing: one wrong level registration and two registrations errors which placed the scan in the wrong orientation over the vertebra. For both modalities, there were no major breaches exceeding the pedicle cortex > 2 mm (grade C, D or E); Of the 69 sCT-guided screws, 59 (86%) were grade A and 10 (14%) grade B. For the 68 CT-guided screws, 47 (69%) were grade A and 21 (31%) grade B. A chi-square test showed this to be significantly different (*p* = 0.022) in favor of sCT. Average distance (± SD) between intraoperative and postoperative screw positions was 2.3 mm ± 1.5 mm for sCT-guided screws, and 2.4 mm ± 1.8 mm in CT-guided screws, which was not significantly different (*p* = 0.78). The maximum angulation (± SD) between intraoperative and postoperative screw positions was on average 3.8 ± 2.5° for sCT and 3.9 ± 2.9° for CT, which was also not significantly different (*p* = 0.75).

## Discussion

This is a safety and accuracy study of a completely radiation-free spinal navigation workflow, consisting of pre-operative MRI-based sCT acquisition and a radiation-free spinal navigation technique. Preoperative imaging was performed with a scoliosis-specific MRI-protocol, providing both conventional T2-weighted MRI and MRI for sCT reconstruction in one short scanning session. The workflow with sCT was compared to the current ‘gold standard’ imaging in spinal navigation: high-resolution thin-slice CT. It was shown that the use of the sCTs is feasible and safe in a cadaveric model, with no severe cortical breaches and no significant differences in distance and angulation between planned and postoperative screw positions compared to CT.

Radiation exposure is a significant problem for scoliosis patients: follow-up and treatment currently heavily rely on it, even though we know about the harmful effects. Considering that the cumulative radiation dose of radiographic follow-up and pre- and postoperative 3-D imaging in case of surgery becomes even higher, iatrogenic cancer is a threat that should be considered. Therefore, efforts are being made to keep the radiation dose as low as reasonably achievable (ALARA principle). At the same time, surgical innovations (that typically rely on radiation) are adopted by surgeons to keep the neurological complication rate low. Modern spinal navigation surgery is mostly performed using images with less radiation than conventional CT. Noto et al. (2024), showed that dual-source CT leads to 75% less radiation exposure than conventional CT, with similar thoracic pedicle screw accuracy [19]. While it can be discussed whether the radiation dose is ethically acceptable relative to surgical risks reduction, still many pediatric deformity surgeons rely on free-hand pedicle screw instrumentation because of concerns of radiation exposure and absence of radiation-free alternatives.

The workflow of this study is promising: it is comparable to CT in terms of usability, safety and accuracy and completely radiation-free. The medial breach rate, angulation and distance from the planned screws were in-line with previous studies on navigated or robot-assisted pedicle screw instrumentations in the literature. A geometric model of the spinal anatomy by Rampersaud et al. (2001), showed satisfactory screw positions allowed for maximal rotational errors of less than 5° [20]. In a review by Mason et al. (2014), fluoroscopy showed accurate screw placement in 50.8% and 75.9% at thoracic and lumbosacral levels respectively (misplaced screws defined as any degree of pedicle cortex perforation). In the same review, 3-D fluoroscopic navigation showed screw accuracy of 93.2% and 96.7% at thoracic and lumbosacral levels, respectively [21]. We observed an error of 6.9° ± 3.9° between the planned screw trajectories on CT and sCT. However, within the same modality, the actual error between the planned and the postoperative position was 3.9 ± 2.9° for CT and 3.8 ± 2.5° for sCT, which is in the acceptable error range described by Rampersaud et al. (2001) [20]. Therefore, while the variability of manual screw planning on both modalities is probably larger, the accuracy of modalities stayed below the rotational error as earlier described. In comparison with a study by Nottmeier et al., where 951 screws were placed with 3-D image guidance (either preoperative or intraoperative CT), our rate of small medial breaches (grade B) was higher (14% versus 3.3%) [22]. We think this can be explained by the fact that we did not place actual pedicle screws but projected virtual screws with standardized sizes over k-wires. Whereas screws may be centralized by the cortex, virtual screws do not, possibly resulting in a higher percentage of grade B. This limitation, however, has no impact on the primary research question on safety and accuracy of sCT *versus* CT in this study. Moreover, in our experiment sCT-guidance resulted in significantly more virtual screws with grade A than CT-guidance.

This cadaver experiment seems to indicate that the radiation-free workflow with the commercially available system used for intraoperative registration (point-cloud surface matching) is safe for clinical introduction and takes only a few minutes per vertebra. Practically, however, for pediatric scoliosis surgery most clinicians are used to conventional weight-bearing preoperative images for level selection, and to use fluoroscopy to confirm correct-level surgery. Furthermore, manual point-cloud registration for all levels could lead to prolonged anesthesia. As alternatives, the integration of scoliosis-specific MRI, including MRI-based sCT, with other radiation-free techniques still has to be explored. Examples are automatic, machine-vision surface matching or the use of patient-specific 3-D printed guides. A study by Cool et al. (2021) showed that with the use of low-dose CT-scans, pedicle guides could be created with a 100% accuracy of pedicle screw placement [23]. Another potential advantage of the MRI-protocol in the future is that the T2w images of the spinal canal could be combined with the osseous segmentations of the sCT images into one image. That would allow for navigation with visualization of the spinal cord relative to the pedicles. This could be useful for screening for neural abnormalities and pre-operative neural risk assessment of intraoperative neuromonitoring loss in scoliosis patients using the Spinal Cord Shape Classification System (SCSCS) class [24, 25].

*Limitations*

An important inherent limitation of cadaveric experiments is that the real-life surgery setting and patients are different. The MRIs of relatively old cadavers without scoliosis and with osteoporotic spines are different than in vivo imaging of pediatric scoliosis. MRI scans of cadavers at room temperature instead of physiological body temperature may result in different MRI contrasts. We did not observe clear differences between the image quality of the automatic sCT reconstructions and clinically obtained sCT scans of real scoliosis patients. While osteoporotic effects were visible in the lateral parts of the transverse processes on both imaging modalities, surface matching was possible at all levels. In the 3-D reconstructions in the software, it was noticed that MRI-based sCTs had a visually more smoothened surface of the lamina’s than the high-resolution CT images. This effect could be caused by the MRI reconstruction algorithm smoothening gaps. This did not result in significant differences in safety or accuracy. In our experience, the surface of the cortical bone on sCT scans of adolescents is generally smoother, on CT as well as sCT.

In this study, navigated pedicle screw placement was performed by experienced spine deformity surgeons. Although the aim was to solely rely on navigation, we realize that these spine surgeons may unintentionally use their experience, potentially resulting in an overestimation of the accuracy of navigation. However, as in the clinical setting, navigation will also mostly be used by experienced surgeons, it still represents the added value of assistance by spinal navigation for pedicle screw placement when comparing sCT to CT. To assess the absolute value of sCT alone, we would recommend a future study with experienced spine surgeons performing in similar patients both freehand and sCT-based navigated pedicle screw placement. To explore if MRI-based sCT can be used for spinal navigation in pediatric scoliosis patients, a logical next step would be a study that uses this technique in real-life patients. We think the current cadaveric study indicates that such a study can be performed safely.

## Conclusion

In conclusion, this experimental cadaveric study demonstrates no significant difference of sCT-based spinal navigation compared to conventional CT in terms of safety (grade A or B) and accuracy (distance and angulation) of thoracic and lumbar pedicle screw placement. Synthetic CT based spinal navigation in pediatric spinal deformity surgery will reduce the perioperative radiation exposure. To prove the accuracy and safety in real-life scoliosis patients, this technique should be further explored in pediatric deformity surgery.

## Data Availability

The data supporting this study are available upon reasonable request from the corresponding author (TS).
